# 3-dimensional surface geometry, optical properties dataset of Scots pine and Norway spruce shoots

**DOI:** 10.1016/j.dib.2025.112420

**Published:** 2025-12-24

**Authors:** Oleksandr Borysenko, Petr Lukeš, Tomáš Hanousek, Lucie Homolová, Růžena Janoutová, Mirjam Uusõue, Steffen Noe, Jan Pisek

**Affiliations:** a University of Tartu, Tartu Observatory, Observatooriumi 1, 61602 Tõravere, Tartumaa, Estonia; b Global Change Research Institute of the Czech Academy of Sciences, Bělidla 4a, 603 00 Brno, Czech Republic; c Department of Geography, Faculty of Science, Masaryk University, Kotlářská 2, 611 37 Brno, Czech Republic; d Chair of Forest and Land Management and Wood Processing Technologies, Institute of Forestry and Engineering, Estonian University of Life Sciences, 51006 Tartu, Estonia

**Keywords:** Shoot structure, GOM photogrammetry, Radiative transfer, 3D virtual plant canopy, Reflectance and transmittance factors, Norway spruce, Scots pine

## Abstract

Conifer shoots possess highly complex geometrical structures at a very fine spatial resolution. Accurately characterizing the full architecture of a conifer shoot, which influences how radiation is scattered, has proven challenging. Previous radiative transfer models for coniferous stands have represented these structures in a relatively simplified or coarse manner. This paper presents a dataset that can be used for up-scaling of needle to shoot optical properties and studying the influence of detailed three-dimensional (3D) structure of shoot to light scattering within tree crown. The dataset includes 3D structural information as well optical properties of needles and twigs for 27 shoots of two conifer species present in both locations (3 shoots per species and position in the crown) - Scots pine (*Pinus sylvestris* L.) and Norway spruce (*Picea abies* L. *Karst.*). The samples were collected on 22nd April 2024 in Rájec, the Czech Republic and 17th September 2024 in Järvselja, Estonia. Subsequently blue light 3D photogrammetry scanning technique was used to obtain their high-resolution 3D point cloud representations. Reflectance and transmittance measurements of needles were obtained using a spectroradiometer and an integrating sphere. For each of these samples, the dataset comprises a photo of the sampled shoot, obtained 3D surface reconstruction, and optical properties of conifer needles and twigs (hemispherical-conical reflectance and transmittance factors) in the spectral range of 400-2000 nm. A detailed 3D representation of needle shoots, when combined with radiative transfer modeling, may offer a means to study and compensate for inaccuracies in the measurement of needle optical properties and to enhance the assessment of shoot scattering characteristics.

**Table:** Specifications Table

Subject	Earth & Environmental Sciences
Specific subject area	Anatomy; ecophysiology; conifer shoot; radiative transfer modeling; remote sensing
Type of data	Processed data in Tables (.csv), Photos (.jpg), Geometry files (.obj)
Data collection	*The Pinus sylvestris* L. and *Picea abies* (L.) *Karst.* branches were collected from both the upper and lower parts of tree crowns in the Czech Republic in April 2024 and Estonia in September 2024. The branches were carefully placed in a cooling box to protect the shoots and preserve their structural integrity. In the laboratory, samples—representing current-year, one-year-old, and two-year-old growth - were analyzed. The shoots were scanned with an industrial non-contact 3D scanner GOM Scan 1 (MV 200 version) (Carl Zeiss GOM Metrology GmbH, Germany). Reflectance and transmittance spectra of needles were measured using an ASD FieldSpec® 4 spectroradiometer (Malvern Panalytical Ltd., Malvern, UK).
Data source location	Localization: Rájec Country: Czech Republic Latitude and longitude: 49° 26′ 44.88″ N, 16° 41′ 47.76″ E Localization: Kanice Country: Czech Republic Latitude and longitude: 49° 15′ 56.68″ N, 16° 40′ 44.86″ E Localization: The SMEAR Estonia station Country: Estonia Latitude and longitude: 58° 16′ 17.04″ N, 27° 16′ 13.08″ E
Data accessibility	Repository name: Mendeley Data identification number: 10.17632/h39f9t7fjg.1 Direct URL to data: https://data.mendeley.com/datasets/h39f9t7fjg/2

## 1 Value of the Data

• The dataset provides detailed 3D shoot models and spectral libraries for Norway spruce and Scots pine, the two most widespread conifer species in Europe. This makes it a valuable resource for applications in remote sensing, ecosystem characterization, and climate-related research where species-specific optical and structural data are required.
• The spectral and structural information included in the dataset supports the development and calibration of radiative transfer models. In particular, it enables more accurate characterization of the shortwave radiation regime of vegetation, which is fundamental for simulating energy and matter exchange between vegetation and the atmosphere.
• The high-resolution 3D shoot representations allow researchers to explore methods for scaling optical properties from the shoot level to the canopy level. Such information can serve as a reference for improving measurement techniques and upscaling strategies in studies of coniferous forest canopies.
• The dataset includes 3D models that capture fine details of needle arrangement, shoot structure, and overall morphology. These data can be applied to analyses of shoot-level traits, supporting investigations of structural variability and its potential influence on ecological processes and tree functioning.
• Beyond forestry and ecology, the conifer shoot representations can be incorporated into the creation of virtual trees and forest scenes. They can enhance environmental simulations, visualizations, and educational tools by providing realistic structural detail that improves the authenticity of rendered vegetation.

## 2 Background

This dataset was initially compiled to assess whether the integration of realistic shoot models within high-resolution 3D radiative transfer simulations—and the direct use of measured optical properties of needles and twigs—could improve modeling accuracy and efficiency compared to conventional goniometer-based approaches. The current data article extends the value of the conducted research by making the dataset publicly accessible and supporting a wider range of research applications and contributing to the advancement of 3D radiative transfer modeling in coniferous forest ecosystems. While the original study focused on Norway spruce (*Picea abies* (L.) *Karst.*), the dataset has since been expanded to include Scots pine (*Pinus sylvestris* L.) as well. These two species are recognized as the most widespread conifers in Europe, based on the spatially representative Level II monitoring network of the International Co-operative Programme on Assessment and Monitoring of Air Pollution Effects on Forests (ICP Forests; www.icp-forests.net), which employs a 16 km grid system [1].

## 3 Data Description

**Fig. 1:**
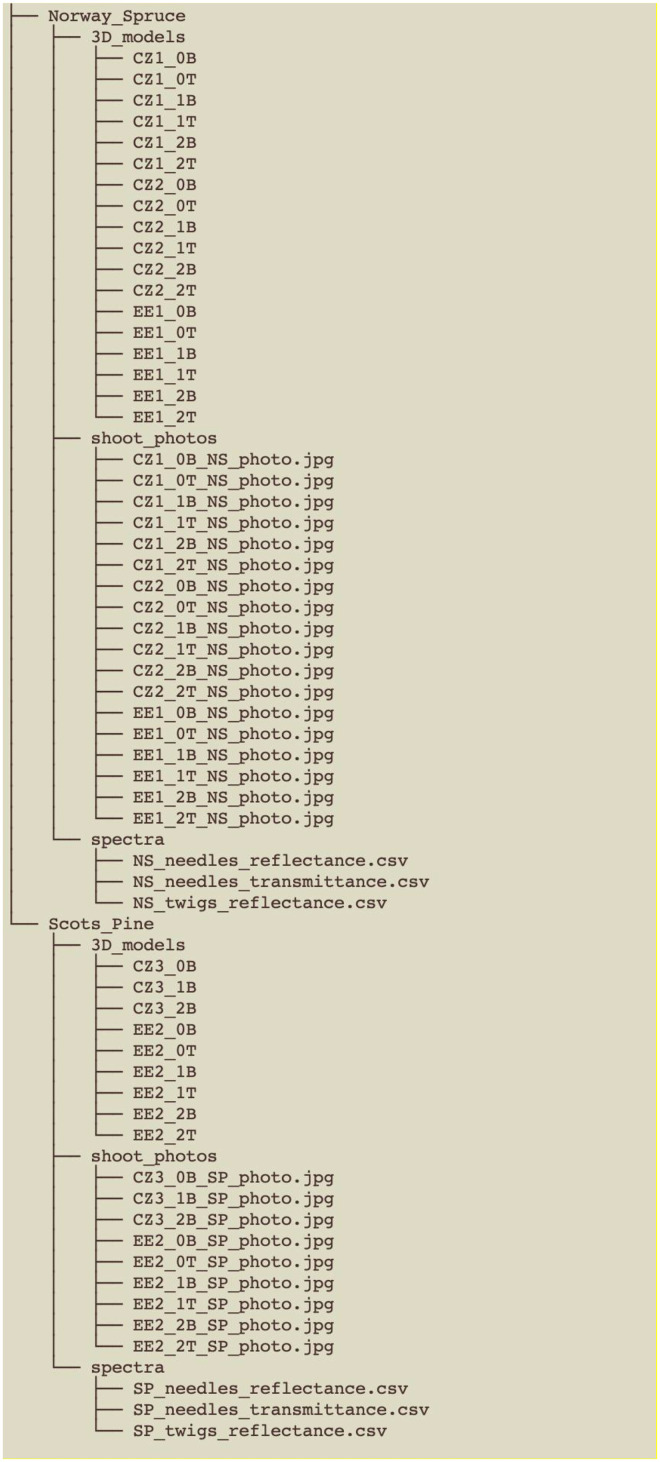
Dataset files and folders structure.

The dataset can be downloaded from https://data.mendeley.com/datasets/h39f9t7fjg/2. It consists of a single compressed folder. Once unpacked, the data are organized as shown in Fig. 1. Two main folders, each corresponding to a tree species, contain three subfolders. The first folder contains 3-dimensional surface geometry models of shoots for the given species (.obj format). The second subfolder contains a photo (.JPG format) of each scanned sample shoot. The third subfolder includes the optical properties of the species’ conifer needles and twigs, specifically the hemispherical-conical reflectance (HCRF) and transmittance factors (HCTF) data (.csv format). In the file names, the first two letters indicate the country code for the given site (CZ or EE), followed by the site number (1, 2, 3). Next is the shoot age (0, 1, 2 years) and the shoot position (bottom (B) or top (T) of the crown).

## 4 Experimental Design, Materials and Methods

### 4.1 Study sites and sample collection

This dataset combines data from two field campaigns conducted in the Czech Republic and Estonia. Each campaign produced detailed 3D structural scans and measurements of needle and twig optical properties.

The field campaign in the Czech Republic covered two sites: Rájec and Kanice. Rájec is a temperate forest dominated by ∼40-year-old Norway spruce (*Picea abies* (L.) H. Karst), with a mean tree height of 25.38 ± 4.19 m and a mean DBH of 51.82 ± 14.45 cm. The Kanice site is characterized by Scots pine (*Pinus sylvestris* L.) with a mean tree height of 18.28 ± 0.90 m and mean DBH of 17.96 ± 3.55 cm. On 22 April 2024, branches were collected from the upper and lower crowns of two dominant Norway spruce trees and one Scots pine tree all from the lower crowns. In the laboratory, three shoot age classes (current-year, one-year-old, and two-year-old) were sampled from both crown positions (a total of 15 samples), along with paired samples for destructive optical measurements. Following reflectance measurements, the shoots were transported in a cooling box to Estonia and 3D-scanned within a few days.

The Estonian site, SMEAR Estonia, is a hemiboreal forest ecosystem co-dominated by birch species (*Betula pendula* Roth and *B. pubescens* Ehrh.) and coniferous species, mainly Norway spruce (*Picea abies* (L.) H. Karst) and Scots pine (*Pinus sylvestris* L.) [2]. On 17 September 2024, branches were collected from the upper and lower crowns of a suppressed Norway spruce (∼10 m tall) and Scots pine (∼17 m tall) using a scaffolding tower and telescopic pruner. As at the Czech site, three shoot age classes were sampled from each crown position (12 samples total), along with twin samples for optical analyses.

### 4.2 Measurement of 3D shoot structure

**Fig. 2:**
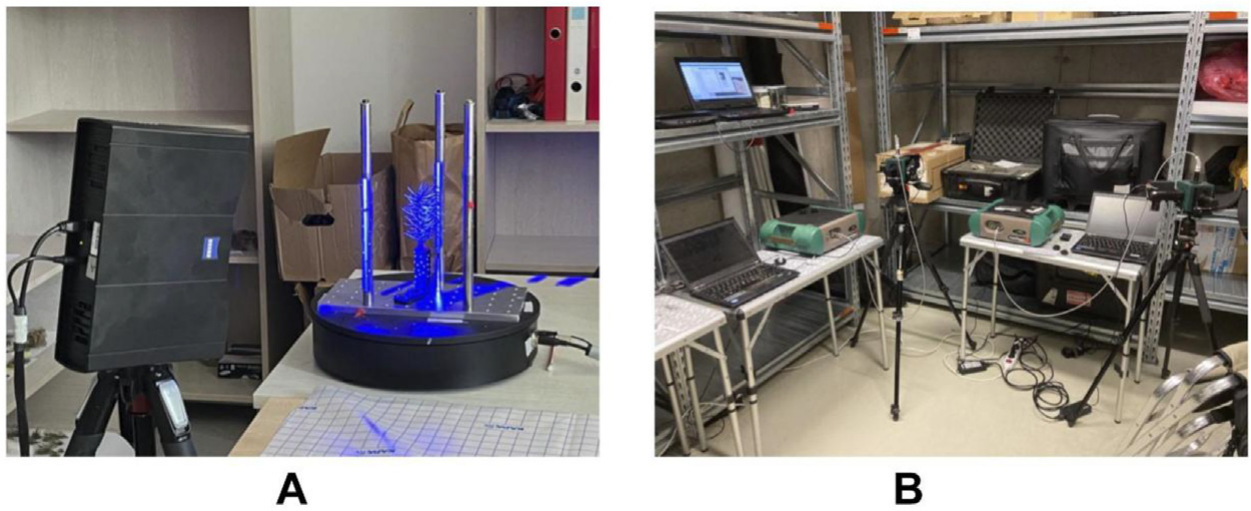
Measuring set ups for the blue light 3D scanning (A), reflectance and transmittance (B).

The shoots were scanned using an industrial non-contact 3D scanner, the GOM Scan 1 (MV 200 version) (Carl Zeiss GOM Metrology GmbH, Germany). A brief overview of the scanning process is provided here; for a detailed description, see [3]. The scanner employs structured narrow-band blue light and two cameras operating on the stereo camera principle. Our setup included an automated rotation table, the GOM ROT 350, with needle shoots mounted upright at the center of the table (Fig. 2A).

Using GOM Inspect software, we pre-set the rotation step, and the entire scanning procedure was automated. Within approximately 5 min, up to 60 scans were acquired per shoot, capturing a full 360° view with a nominal point spacing of 0.06 mm. Depending on shoot size and morphological complexity, between 35 and 101 scans were collected per sample. The final number of scans was determined by visually inspecting the point clouds to ensure complete surface coverage without significant gaps.

**Fig. 3:**
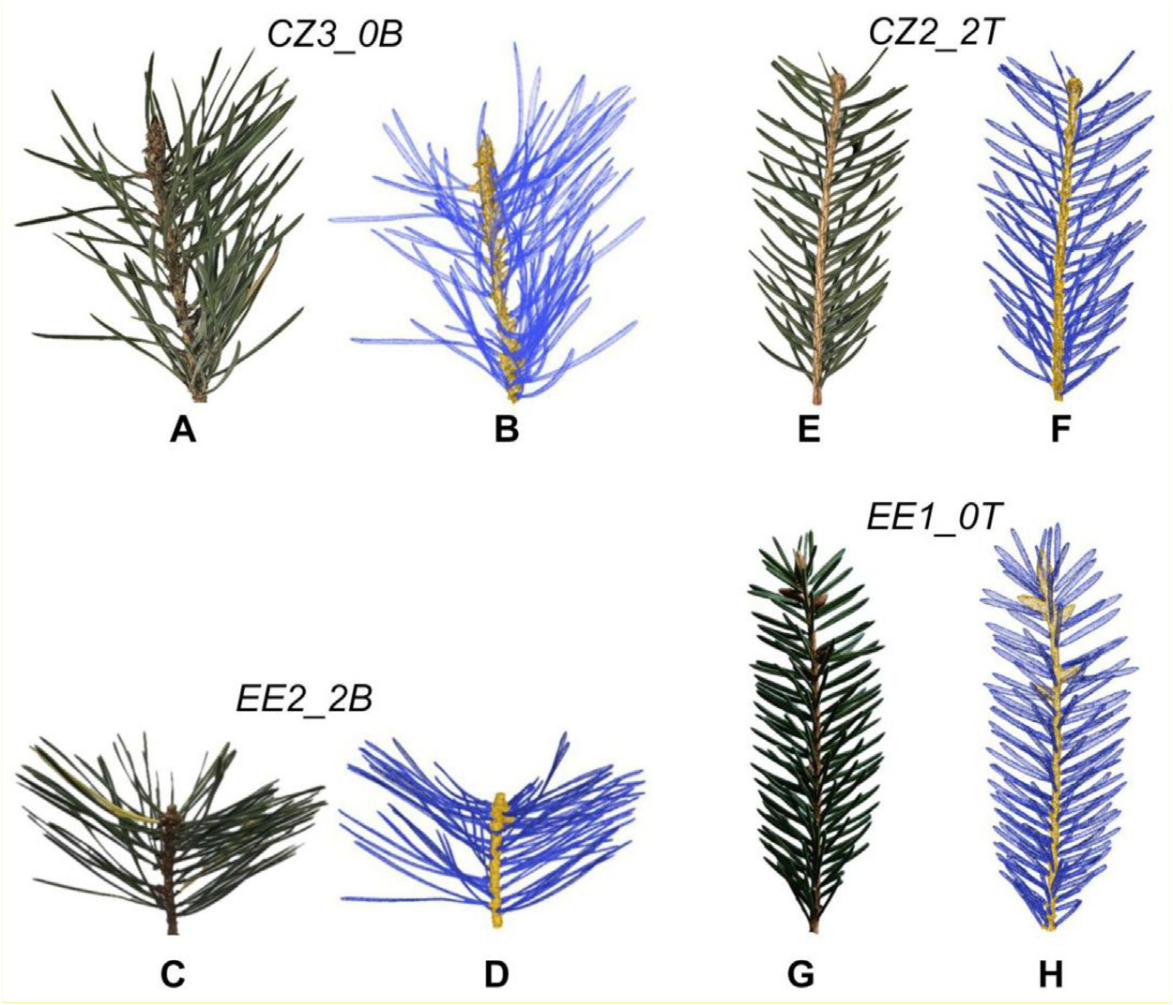
Comparison between actual shoots and corresponding reconstructed 3D surfaces for sampled Scots pine shoots from the Czech Republic (A, B) and Estonia (C, D), and Norway spruce shoots from the Czech Republic (E, F) and Estonia (G, H).

After scanning, any remaining gaps in the models were closed manually or semi-automatically using interactive tools in GOM Inspect. The resulting point clouds for individual shoots contained between 0.3 million and 1.0 million points. Meshes were then exported in STL format and processed in MeshLab [4] to separate the needle and twig components of each shoot. Subsequently, both components were saved in OBJ format (Fig. 3).

### 4.3 Measurement of optical properties

**Fig. 4:**
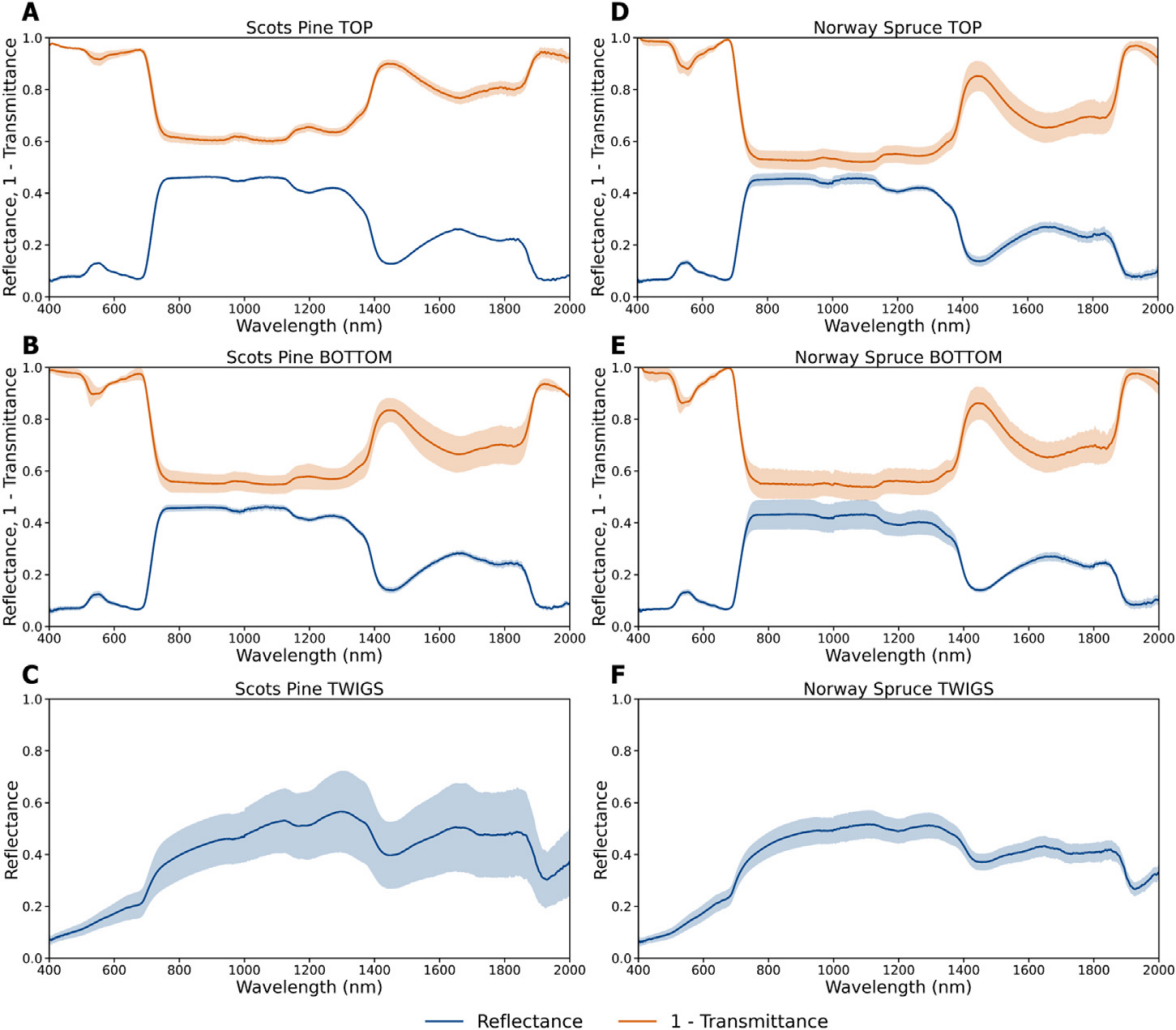
Mean and standard deviation of reflectance (blue) and transmittance (orange) of all pine (A, B) and spruce (D, E) needles from top (A, D) and bottom (B, E) branches of the trees and mean and standard deviation of reflectance of twigs from pine (C) and spruce (F) samples.

The optical properties of conifer needles, specifically the hemispherical-conical reflectance (HCRF) and transmittance (HCTF) factors [5], were measured using an ASD FieldSpec® 4 spectroradiometer (Malvern Panalytical Ltd., Malvern, UK) coupled with an ASD RTS-3ZC integrating sphere. In Estonia, a single spectroradiometer (serial number: 18,119) and integrating sphere were used, whereas in the Czech Republic, two separate spectroradiometer–sphere systems operated in parallel—one dedicated to reflectance (serial number: 18,119) and the other to transmittance measurements (serial number: 18,118) (Fig. 2B). This dual setup facilitated a more efficient acquisition process and minimized measurement uncertainties associated with changes in the integrating sphere's port configuration, particularly concerning the light source. All measurements were conducted over the 350–2500 nm spectral range with 1 nm spectral resolution, yielding 2151 spectral data points per sample. Due to increased noise levels at the spectral extremes, the analysis was limited to the 400–2000 nm range (Fig. 4).

Due to the small size of individual needle samples, which do not completely fill the sampling port of the integrating sphere, we employed a gap-fraction measurement approach. In this method, needles are arranged in a custom sample holder, resulting in inevitable gaps between them that must be accounted for during post-processing of the optical measurements [6]. Reflectance measurements of twigs were also carried out using the same sampling protocol and gap-fraction correction method.

Gap fractions were estimated using two complementary methods:

1) Image analysis: Carriers containing the needle samples were scanned using an EPSON V500 Photo flatbed scanner at 600 DPI, producing 16-bit grayscale images. These were converted to binary images using Otsu’s thresholding algorithm. The gap fraction was then quantified as the ratio of white (gap) to black (needle) pixels within the area illuminated by the light source.
2) Empirical retrieval from reflectance and transmittance spectra, following the method described by [7]. For reflectance, the gap fraction (GF_R) was estimated based on the measured uncorrected reflectance at 800 nm:

For transmittance, the gap fraction (GF_T) was derived from the measured uncorrected transmittance at 450 nm:

$$\text{GF}\_T=\left(T_{450}-0.03\right)/0.93$$

Here, R₈₀₀ is the uncorrected reflectance at 800 nm, and T₄₅₀ is the uncorrected transmittance at 450 nm.

There was a moderately strong correlation between image-derived and wavelength-retrieved gap fraction values (R² = 0.766, RMSE = 0.17); however, the image-derived values were, on average, 33 % higher than those retrieved from the wavelength method (results not shown). Since the wavelength-derived gap fractions produced less variability in the resulting spectra, we opted to use this method. Additionally, because the transmittance spectra still exhibited negative values around the red spectral region (∼550 nm), we adjusted the retrieved gap fraction values for transmittance samples to ensure positive values in this region.

We measured all data in raw digital number (DN) values, recording the sample DNs and white reference DNs separately. The uncorrected Hemispherical-Conical Reflectance Factor (HCRF_orig_) and Hemispherical-Conical Transmittance Factor (HCTF_orig_) were calculated as follows:

$$\text{HCR}F_{\text{orig}}=\left(\left(DN_{\text{Rsample}}-DN_{\text{dark}}\right)*R_{\text{WR}}\right)/\left(DN_{\text{WR}}-DN_{\text{dark}}\right)$$

$$\text{HCT}F_{\text{orig}}=\left(\left(DN_{\text{Tsample}}-DN_{\text{dark}}\right)*R_{WR}\right)/\left(DN_{\text{WR}}-DN_{\text{dark}}\right),$$

where DN_Rsample_ is a DN value of sample measured in reflectance mode, DN_dark_ is a DN value of sphere interior without light source and sample, DN_WR_ the DN value of spectralon white reference panel, R_WR_ true reflectance of calibrated spectralon panel and DN_Tsample_ the DN value of sample measured in transmittance mode.

Final gap-fraction corrected needle HCRFs and HCTFs were calculated as:

$$\text{HCR}F_{\text{corr}}=\text{HCR}F_{\text{orig}}/\left(1-\text{GF}\_R\right)$$

$$\text{HCT}F_{\text{corr}}=\left(\text{HCT}F_{\text{orig}}-\left(R_{\text{sphere}}*\text{GF}\_T\right)\right)/\left(1-\text{GF}\_T\right)$$

where HCRF_corr_ is a gap-fraction corrected HCRF, HCRF_orig_ the original uncorrected HCRF, GF_R the gap fraction of the sample in reflectance mode, HCTF_corr_ the gap-fraction corrected HCTF, R_sphere_ the sphere wall reflectance calibration file and GF_T the gap fraction of the sample in transmittance mode.

## Limitations

The studied conifer shoots come from two dominant (Czech Rep.) or non-dominant (Estonia) trees. The surface models of conifer shoots presented in the dataset may not fully capture the complete range of architectural shoot types present in the corresponding plots. The uncertainties in the reported optical property measurements arise from several sources. Instrumental variability, particularly between different integrating spheres, introduces reflectance discrepancies ranging from ±0.005 to ±0.044 DHRF, as shown by [8]. Corrections for needle gap fraction contribute an estimated ±5–10 % reflectance error due to methodological assumptions [7]. Calibration uncertainty of Spectralon reference panels contributes an additional ±2–5 %. Errors related to directional measurement geometry—especially fiber distance and zenith/azimuth alignment—can introduce ±2–5 % variability. Finally, within-sample reproducibility, even with replicate measurements, contributes a remaining ±1–3 % uncertainty.

The sampling design introduces several limitations that should be acknowledged. First, the shoots were collected from only three dominant trees at the Czech site and from two non-dominant trees at the Estonian site. This restricted sampling does not fully represent the structural and physiological variability that may occur across different canopy positions, tree ages, competitive statuses, or microsite conditions. As a result, the shoot surface models included in the dataset likely capture only a subset of the architectural diversity present within the stands, particularly regarding branching patterns, needle density, and shoot morphology. Moreover, the limited number of shoot age classes and the lack of replication across multiple individuals further constrain the generalizability of the findings.

## Ethics Statement

The authors declare that this work did not involve human subjects nor animal experiment nor data collected from social media platforms. They have read and followed the ethical requirements for publication in Data in Brief journal.

## Credit Author Statement

**Oleksandr Borysenko**: Methodology, Software, Visualization, Investigation, Data Curation, Writing – original draft preparation; **Petr Lukeš**: Methodology, Software, Visualization, Investigation, Data Curation, Writing – original draft preparation; **Tomáš Hanousek**: Methodology, Writing – review & editing; **Lucie Homolová**: Methodology, Investigation, Data Curation, Writing – review & editing; **Růžena Janoutová**: Methodology, Software, Visualization, Investigation, Writing – review & editing, Funding acquisition; **Mirjam Uusõue**: Investigation; **Steffen Noe**: Funding acquisition, Writing – review & editing; **Jan Pisek**: Conceptualization, Methodology, Writing – review & editing, Funding acquisition.

## Data Availability

Mendeley DataA dataset of detailed 3-dimensional surface geometry, optical properties of Scots pine and Norway spruce shoots (Original data).
